# *Porphyromonas gingivalis*-Induced Neuroinflammation in Alzheimer’s Disease

**DOI:** 10.3389/fnins.2021.691016

**Published:** 2021-10-14

**Authors:** Ingar Olsen

**Affiliations:** Department of Oral Biology, Faculty of Dentistry, University of Oslo, Oslo, Norway

**Keywords:** amyloid-beta, tau, microglia, cathepsin B, protein kinase R, lipopolysaccharide, gingipains

## Abstract

“Chronic” periodontitis and its keystone pathogen *Porphyromonas gingivalis* have repeatedly been associated with Alzheimer’s disease (AD). Pathological hallmarks in AD are brain accumulations of amyloid-beta and neurofibrillary tangles consisting of aggregated and hyperphosphorylated tau. In addition, neuroinflammation induced by *P. gingivalis* has increasingly been recognized as a factor in the pathogenesis of AD. The present mini-review discusses possible mechanisms for the induction of neuroinflammation by *P. gingivalis* in AD, involving factors such as pro-inflammatory mediators, amyloid-beta, tau, microglia, cathepsin B, and protein kinase R. Inflammagens of *P. gingivalis* such as lipopolysaccharide and gingipains are also discussed.

## Introduction

“Chronic” periodontitis is a disease affecting the supporting tissues of the teeth. Untreated, it may end with tooth loss. It is a widely prevalent disease in adults all over the world (Eke et al., 2016) and has in several reports been associated with Alzheimer’s disease (AD) (for a review, see Olsen, 2021). *Porphyromonas gingivalis*, which is considered a keystone bacterium in “chronic” periodontitis (Socransky et al., 1998; Darveau et al., 2012; Hajishengallis et al., 2012), has been detected in the brains of subjects with AD together with its toxic proteases—gingipains (Dominy et al., 2019). Also, *P. gingivalis* DNA was found in AD brains and cerebrospinal fluid of clinical AD patients. In other studies, *P. gingivalis* lipopolysaccharide (LPS) was detected in human AD brains and in the brains from transgenic mice serving as AD models (Poole et al., 2013; Ishida et al., 2017).

Several animal studies have indicated that *P. gingivalis* can induce neuroinflammation in the brain of AD patients (see later), and neuroinflammation has increasingly been suggested to have a substantial role in the progression of the neuropathological changes taking place in AD (Ilievski et al., 2018). This mini-review will deal with neuroinflammation in AD induced by *P. gingivalis* and possible mechanisms for this induction.

## Neuroinflammation and *Porphyromonas gingivalis*

Alzheimer’s disease is our commonest neurological disease characterized by cognitive decline and accumulation of amyloid-beta (Aβ) plaques and neurofibrillary tangles (NTFs). Neuroinflammation has increasingly been considered as another hallmark of AD. *P. gingivalis*-LPS-induced neuroinflammation was proposed to play an important role in the cognitive impairment of C57BL/6 mice (Zhang et al., 2018). Hu et al. (2020) found that periodontitis induced by *P. gingivalis*-LPS promoted neuroinflammation by activating the Toll-like receptor 4/nuclear factor-kappa B signaling pathway and was associated with learning and memory impairment in Sprague-Dawley rats. In another study, *P. gingivalis* periodontal infection was proposed to cause cognitive impairment by releasing pro-inflammatory cytokines such as tumor necrosis factor-alpha (TNF-α), interleukin (IL)-6, and IL-1β in the brain tissues of middle-aged mice (Ding et al., 2018). Similarly, Memedovski et al. (2020) reported that *P. gingivalis* LPS induced classical and alternative activation of rat brain microglia with concomitant release of cytokines and chemokines.

## Amyloid-Beta and *Porphyromonas gingivalis*

Amyloid-beta is known to be an activator of microglia. On the one hand, microglia can release inflammatory mediators such as inflammatory cytokines, complement components, chemokines, and free radicals that all contribute to Aβ production and accumulation. On the other hand, microglia can play a beneficial role in generating anti-Aβ antibodies and stimulating the clearance of Aβ plaques (Cai et al., 2014). According to these authors, a vicious cycle of inflammation occurs between Aβ accumulation, activated microglia, and microglia inflammatory mediators, which promotes Aβ deposition and neuroinflammation. This idea diverges from the general notion that Aβ production and neuroinflammation are independent processes.

Nie et al. (2019) reported that chronic exposure to *P. gingivalis* LPS led to the accumulation of Aβ in the brain of middle-aged mice. Such exposure also induced peripheral Aβ accumulation in inflammatory monocytes/macrophages. This suggested that monocytes/macrophages can serve as a circulating pool of Aβ in patients with periodontitis. Similarly, Leira et al. (2019) reported that *P. gingivalis*-induced LPS in periodontitis produced increased serum levels of Aβ peptides. In mice, oral *P. gingivalis* infection caused brain colonization and increased production of the amyloid plaque component Aβ_1__–__42_ (Dominy et al., 2019). Importantly, the neuroinflammation established by *P. gingivalis* in the mice could be reduced by gingipain inhibition.

## Tau Protein and *Porphyromonas gingivalis*

As mentioned, NFTs are created from hyperphosphorylated tau—a protein that stabilizes microtubules (for a review, see Kinney et al., 2018). In AD, hyperphosphorylated tau is removed from microtubules resulting in a collapse of the microtubule structure and thereby disrupted cellular functions for protein trafficking and cellular morphology, formation of tau aggregates, loss of neuronal function, and apoptosis.

There is a clear relationship between *P*. *gingivalis* and tau. Gingipains may cleave procaspase-3 to activate caspase-3 (Urnowey et al., 2006). The latter has been associated with tau phosphorylation (Chu et al., 2017) and tau cleavage (Sandhu et al., 2017). Dominy et al. (2019) found tau to be a target of gingipain proteolysis and suggested that tau pathology in AD brains may be caused by transneural spread of *P. gingivalis*, tau damage by gingipain proteolysis, and activation of human proteases. They also hypothesized that gingipains might be a driver of a compensatory increase in tau production of AD patients.

Tang et al. (2021) confirmed that peripheral *P. gingivalis* infection caused tau hyperphosphorylation, preventing tau from fulfilling its role as a microtubule-stabilizing protein, leaving it to self-assembly. In *P. gingivalis*-injected rats, the severity of phosphorylated tau at the AD-related sites Thr181 and Thr231 and the number of activated astrocytes were greater than in the hippocampus. Also, the levels of IL-1β, IL-6, and TNF-α in the rat serum and hippocampus were increased. Furthermore, the activity of protein phosphatase 2A (PP2A) was significantly inhibited in the hippocampus of these rats. Inhibition of PP2A and application of a PP2A promoter efficiently decreased IL-1β-induced tau hyperphosphorylation in HT-22 cells. Although systemic inflammation was identified as the driver of tau phosphorylation, the specificity of *P. gingivalis* producing this effect was not assessed. Laurent et al. (2018) and Didonna (2020) emphasized tauopathies and neuroinflammatory processes as a vicious circle that works together in the pathogenesis of AD. A link between pro-inflammatory cytokine signaling and hyperphosphorylation of tau has also been reported (Domingues et al., 2017). Of note, usnic acid derivatives were found to inhibit tau aggregation and neuroinflammation (Shi et al., 2020).

A novel mechanism of tau-seed-affected microglia was demonstrated by activation of the NLRP3–ASC inflammasome (Stancu et al., 2019). This inflammasome is an important sensor of innate immunity. Olsen and Singhrao (2016) and Olsen and Yilmaz (2016) reviewed the plausible contribution of specific bacteria playing a role in influencing the activity of the NLRP3 inflammasome in AD progression. *P. gingivalis* was found to have several mechanisms for modulating innate immunity by limiting the activation of the NLRP3 inflammasome. Among them, ATP-/P2X_7_-signaling is associated not only with periodontitis but also with the development of several systemic diseases, including AD.

## Microglia and *Porphyromonas gingivalis*

Hu et al. (2020) observed that *P. gingivalis* LPS-induced periodontitis caused learning and memory impairment in rats through neuroinflammation induced by significant activation of microglia and astrocytes in the brain cortex. Microglia activation may precede tau pathology (Yoshiyama et al., 2007). Memedovski et al. (2020) reported that 18-h *in vitro* stimulation with ultrapure *P. gingivalis* LPS caused classical and alternative activation of rat brain microglia with the release of cytokines and chemokines.

The gingipains Rgp and Kgp have important effects on brain-residing microglia, being responsible for *P. gingivalis*-induced cell migration of microglia and expression of pro-inflammatory mediators by activating the protease-activated receptor 2 (Liu et al., 2017; Nonaka and Nakanishi, 2020). The subsequent activation of phosphoinositide 3-kinase/Akt and mitogen-activated protein kinase/extracellular signal-regulated kinase (ERK) kinase/ERK pathways resulted in cell migration and inflammatory response in microglia. Here, the gingipains of *P. gingivalis* cooperatively contributed to cell migration of microglia toward the infected site (brain) and induction of neuroinflammation after reaching it.

The interaction between genetic factors, microglia, and *P. gingivalis* was reviewed by Olsen and Singhrao (2020). It was suggested that genes for apolipoprotein, clusterin, CD33, triggering receptor expressed on myeloid cells-2, tyrosine kinase binding protein (TYR-OBP), and complement receptors could affect microglia. Most of these genes can also be affected by *P. gingivalis via* its mastering of immune suppression.

## Cathepsin B and *Porphyromonas gingivalis*

Cathepsin B (CatB), a lysosomal cysteine protease, was suggested to have an important role in the initiation of neuroinflammation and neural dysfunction after chronic systemic exposure to LPS from *P. gingivalis* in mice. Thus, Wu et al. (2017) found that such exposure to *P. gingivalis* LPS induced AD-like phenotypes, including microglia-mediated neuroinflammation, intracellular Aβ accumulation in neurons, and reduced learning and memory functions in middle-aged mice in a CatB-dependent manner. As already mentioned, chronic systemic *P. gingivalis* infection induced Aβ accumulation in inflammatory monocytes/macrophages. This occurred *via* activation of CatB/nuclear factor kappa B signaling (Nie et al., 2019). CatB has been suggested as a potential therapeutic target for preventing the initiation and progression of periodontitis-related AD (Nakanishi et al., 2020).

## Protein Kinase R and *Porphyromonas gingivalis*

Protein kinase R (PKR) is a 551 amino acid protein responsible for a key part of the defense against bacterial and viral infections in neurons (Dabo and Meurs, 2012; Marchal et al., 2014). This inflammation-associated kinase protein directly phosphorylates several abnormal and disease-modifying residues within tau, such as Thr181, Ser199/202, Thr231, Ser396, Ser404, and Ser409 (Reimer et al., 2021). The PKR-mediated phosphorylations actively dislocate tau from microtubules in cells. Also, PKR overexpression and knockdown increased and decreased, respectively, tau protein and mRNA levels in cells. It was noteworthy that acute encephalopathy in wild-type mice, induced by intracranial Langat virus infection, resulted in robust inflammation and PKR upregulation, which was followed by abnormally phosphorylated full-length and truncated tau. PKR can be capable of triggering pathological modification of tau independent of other kinases after brain inflammation. This might be the initial pathological seed in tauopathies such as AD and in chronic encephalopathy with severe inflammation. PKR inhibition reduced phosphorylation of soluble tau in the brain of transgenic rTg4510 tau mice (Reimer et al., 2021). Inhibition of PKR also prevented long-term potentiation and memory impairment in AD mouse models (Hwang et al., 2017). Furthermore, PKR inhibition reduced neuronal loss, motor deficits, and memory deficits in mice models of AD (Mouton-Liger et al., 2015; Segev et al., 2015; Reimer et al., 2021).

A direct relationship between *P. gingivalis* and PKR has not yet been demonstrated. However, PKR, a ubiquitously expressed serine–threonine kinase, is activated by indirect binding to bacterial LPS or pro-inflammatory cytokines such as TNF-α, IL-1, and interferon-gamma (for a review, see Reimer et al., 2021). PKR directly regulates tau, and activation of PKR has been associated with different tauopathies such as AD, Parkinson’s disease, and Huntington’ disease (Peel et al., 2001; Chang et al., 2002; Bando et al., 2005; Paquet et al., 2012; Lourenco et al., 2013; Ma et al., 2013). Because PKR is activated indirectly by LPS and specific cytokines, this could contribute to the correlation of “chronic” periodontitis and *P. gingivalis* brain levels with AD (Reimer et al., 2021). Bacterial infections and inflammation could also make neurons vulnerable to degeneration and thus initiate the onset of neurodegenerative diseases such as AD (Deleidi and Isacson, 2012). In this situation, activated PKR could initiate abnormal tau phosphorylation.

## Concluding Remarks

Neuroinflammation seems to have a substantial role in the pathogenesis of AD. This supports neuroinflammation as a third disease hallmark of the disease. *P. gingivalis* with its inflammagens, gingipains, and LPS, both detected in the brains of AD subjects, could be major factors inducing neuroinflammation. *P. gingivalis* particularly affects Aβ, tau, microglia, CatB, and possibly PKR. A vicious cycle of inflammation probably occurs between several of these players where the interaction is complex and not yet fully understood.

Although not specifically related to *P. gingivalis*, PKR stands out as an inflammation-associated kinase of particular interest because it is ubiquitously expressed and an important part of the defense against bacterial infections in neurons. It is activated indirectly by LPS and specific pro-inflammatory cytokines and has been linked to AD. PKR is co-localized with abnormally phosphorylated tau in AD brains and directly regulates tau expression. Thus, PKR activated by *P. gingivalis-*induced brain infection/inflammation/pro-inflammatory cytokines may precede tau phosphorylation and thus participate in the etiology of AD. This should be studied.

## Author Contributions

The author confirms being the sole contributor of this work and has approved it for publication.

## Conflict of Interest

The author declares that the research was conducted in the absence of any commercial or financial relationships that could be construed as a potential conflict of interest.

## Publisher’s Note

All claims expressed in this article are solely those of the authors and do not necessarily represent those of their affiliated organizations, or those of the publisher, the editors and the reviewers. Any product that may be evaluated in this article, or claim that may be made by its manufacturer, is not guaranteed or endorsed by the publisher.

## References

[B1] Bando Y. Onuki R. Katayama T. Manabe T. Kudo T. Taira K. (2005). Double-strand RNA dependent protein kinase (PKR) is involved in the extrastriatal degeneration in Parkinson’s disease and Huntington’s disease. *Neurochem. Int.* 46 11–18. 10.1016/j.neuint.2004.07.005 15567511

[B2] Cai Z. Hussain M. D. Yan L.-J. (2014). Microglia, neuroinflammation, and beta-amyloid protein in Alzheimer’s disease. *Int. J. Neurosci.* 124 307–321. 10.3109/00207454.2013.833510 23930978

[B3] Chang R. C. Wong A. K. Ng H. K. Hugon J. (2002). Phosphorylation of eukaryotic initiation factor-2-alpha (eIF2alpha) is associated with neuronal degeneration in Alzheimer’s disease. *NeuroReport* 13 2429–2432. 10.1097/00001756-200212200-00011 12499843

[B4] Chu J. Lauretti E. Praticò D. (2017). Caspase-3-dependent cleavage of Akt modulates tau phosphorylation via GSK3β kinase: implications for Alzheimer’s disease. *Mol. Psychiatry* 22 1002–1008. 10.1038/mp.2016.214 28138159

[B5] Dabo S. Meurs E. F. (2012). dsRNA-dependent protein kinase PKR and its role in stress, signaling and HCV infection. *Viruses* 4 2598–2635. 10.3390/v4112598 23202496 PMC3509664

[B6] Darveau R. P. Hajishengallis G. Curtis M. A. (2012). *Porphyromonas gingivalis* as a potential community activist. *J. Dent. Res.* 91 816–820. 22772362 10.1177/0022034512453589 PMC3420389

[B7] Deleidi M. Isacson O. (2012). Viral and inflammatory triggers of neurodegenerative diseases. *Sci. Transl. Med.* 4:121s3. 10.1126/scitranslmed.3003492 22344685 PMC3982831

[B8] Didonna A. (2020). Tau at the interface between neurodegeneration and neuroinflammation. *Genes Immun.* 21 288–300. 10.1038/s41435-020-00113-5 33011744

[B9] Ding Y. Ren J. Yu H. Yu W. Zhou Y. (2018). *Porphyromonas gingivalis*, a periodontitis causing bacterium, induces memory impairment and age-dependent neuroinflammation in mice. *Immun. Ageing* 15:6. 10.1186/s12979-017-0110-7 29422938 PMC5791180

[B10] Domingues C. da Cruz E. Silva O. A. B. Henriques A. G. (2017). Impact of cytokines and chemokines on Alzheimer’s disease neuropathological hallmarks. *Curr. Alzheimer Res.* 14 870–882. 10.2174/1567205014666170317113606 28317487 PMC5543563

[B11] Dominy S. S. Lynch C. Ermini F. Benedyk M. Marczyk A. Konradi A. (2019). *Porphyromonas gingivalis* in Alzheimer’s disease brains: evidence for disease causation and treatment with small-molecule inhibitors. *Sci. Adv.* 5:eaau3333. 10.1126/sciadv.aau3333 30746447 PMC6357742

[B12] Eke P. I. Wei L. Borgnakke W. S. Thornton-Evans G. Zhang X. Lu H. (2016). Periodontitis prevalence in adults ≥ 65 years of age, in the USA. *Periodontol. 2000* 72 76–95. 10.1111/prd.12145 27501492 PMC8223257

[B13] Hajishengallis G. Darveau R. P. Curtis M. A. (2012). The keystone-pathogen hypothesis. *Nat. Rev. Microbiol.* 10 717–725. 10.1038/nrmicro2873 22941505 PMC3498498

[B14] Hu Y. Li H. Zhang J. Zhang X. Xia X. Qiu C. (2020). Periodontitis induced by *P. gingivalis*-LPS is associated with neuroinflammation and learning and memory impairment in Sprague-Dawley rats. *Front. Neurosc.* 14:658. 10.3389/fnins.2020.00658 32714134 PMC7344110

[B15] Hwang K. D. Bak M. S. Kim S. J. Rhee S. Lee Y. S. (2017). Restoring synaptic plasticity and memory in mouse models of Alzheimer’s disease by PKR inhibition. *Mol. Brain* 10:57. 10.1186/s13041-017-0338-3 29233183 PMC5727890

[B16] Ilievski V. Zuchowska P. K. Green S. J. Toth P. T. Ragozzino M. E. Le K. (2018). Chronic oral application of a periodontal pathogen results in brain inflammation, neurodegeneration and amyloid beta production in wild type mice. *PLoS One* 13:e0204941. 10.1371/journal.pone.0204941 30281647 PMC6169940

[B17] Ishida N. Ishihara Y. Ishida K. Tada H. Funaki-Kato Y. Hagiwara M. (2017). Periodontitis induced by bacterial infection exacerbates features of Alzheimer’s disease in transgenic mice. *NPJ Aging Mech. Dis.* 3:15. 10.1038/s41514-017-0015-x 29134111 PMC5673943

[B18] Kinney J. W. Bemiller S. M. Murtishaw A. S. Leisgang A. M. Salazar A. M. Lamb B. T. (2018). Inflammation as a central mechanism in Alzheimer’s disease. *Alzheimers Dement.* 4 575–590. 10.1016/j.trci.2018.06.014 30406177 PMC6214864

[B19] Laurent C. Buée L. Blum D. (2018). Tau and neuroinflammation: what impact for Alzheimer’s disease and tauopathies? *Biomed. J.* 41 21–33. 10.1016/j.bj.2018.01.003 29673549 PMC6138617

[B20] Leira Y. Iglesias-Rey R. Gómez-Lado N. Aguiar P. Campos F. D’Aiuto F. (2019). *Porphyromonas gingivalis* lipopolysaccharide-induced periodontitis and serum amyloid-beta peptides. *Arch. Oral Biol.* 99 120–125. 10.1016/j.archoralbio.2019.01.008 30665148

[B21] Liu Y. Wu Z. Nakanishi Y. Ni J. Hayashi Y. Takayama F. (2017). Infection of microglia with *Porphyromonas gingivalis* promotes cell migration and an inflammatory response through the gingipain-mediated activation of protease-activated receptor-2 in mice. *Sci. Rep.* 7:11759. 10.1038/s41598-017-12173-1 28924232 PMC5603557

[B22] Lourenco M. V. Clarke Lourenco M. V. Clarke J. R. Frozza R. L. Bomfim T. R. Forny-Germano L. (2013). TNF-α mediates PKR-dependent memory impairment and brain IRS-1 inhibition induced by Alzheimer’s β-amyloid oligomers in mice and monkeys. *Cell Metab.* 18 831–843. 10.1016/j.cmet.2013.11.00 24315369

[B23] Ma T. Trinh M. A. Wexler A. J. Bourbon C. Gatti E. Pierre P. (2013). Suppression of eIF2α kinases alleviates Alzheimer’s disease-related plasticity and memory deficits. *Nat. Neurosci.* 16 1299–1305. 10.1038/nn.3486 23933749 PMC3756900

[B24] Marchal J. A. Lopez G. J. Peran M. Comino A. Delgado J. R. Garcia-Garcia J. A. (2014). The impact of PKR activation: from neurodegeneration to cancer. *FASEB J.* 28 1965–1974. 10.1096/fj.13-248294 24522206

[B25] Memedovski Z. Czerwonka E. Han J. Mayer J. Luce M. Klemm L. C. (2020). Classical and alternative activation of rat microglia treated with ultrapure *Porphyromonas gingivalis* lipopolysaccharide *in vitro*. *Toxins* 12:333. 10.3390/toxins12050333 32438602 PMC7290770

[B26] Mouton-Liger F. Rebillat A.-S. Gourmaud S. Paquet C. Leguen A. Dumurgier J. (2015). PKR downregulation prevents neurodegeneration and β-amyloid production in a thiamine-deficient model. *Cell Death Dis.* 6:e1594. 10.1038/cddis.2014.552 25590804 PMC4669750

[B27] Nakanishi H. Nonaka S. Wu Z. (2020). Microglial cathepsin B and *Porphyromonas gingivalis* gingipains as potential therapeutic targets for sporadic Alzheimer’s disease. *CNS Neurol. Disord. Drug Targets* 19 495–502. 10.2174/1871527319666200708125130 32640970

[B28] Nie R. Wu Z. Ni J. Zeng F. Yu W. Zhang Y. (2019). *Porphyromonas gingivalis* infection induces amyloid-β accumulation in monocytes/macrophages. *J. Alzheimers Dis.* 72 479–494. 10.3233/JAD-190298 31594220

[B29] Nonaka S. Nakanishi H. (2020). Secreted gingipains from *Porphyromonas gingivalis* induce microglia migration through endosomal signaling by protease-activated receptor 2. *Neurochem. Int.* 140:104840. 10.1016/j.neuint.2020.104840 32858090

[B30] Olsen I. (2021). *Porphyromonas gingivalis* may seek the Alzheimer’s disease brain to acquire iron from its surplus. *J. Alzheimer’s Dis. Rep.* 5 79–86. 10.3233/ADR-200272 33681719 PMC7903007

[B31] Olsen I. Singhrao S. K. (2016). Inflammasome involvement in Alzheimer’s disease. *J. Alzheimers Dis.* 54 45–53. 10.3233/JAD-160197 27314526

[B32] Olsen I. Singhrao S. K. (2020). Interaction between genetic factors, *Porphyromonas gingivalis* and microglia to promote Alzheimer’s disease. *J. Oral Microbiol.* 12:1820834. 10.1080/20002297.2020.1820834 33062201 PMC7534375

[B33] Olsen I. Yilmaz Ö (2016). Modulation of inflammasome activity by *Porphyromonas gingivalis* in periodontitis and associated systemic diseases. *J. Oral Microbiol.* 8:30385. 10.3402/jom.v8.30385 26850450 PMC4744328

[B34] Paquet C. Mouton-Liger F. Meurs E. F. Mazot P. Bouras C. Pradier L. (2012). The PKR activator PACT is induced by Aβ: involvement in Alzheimer’s disease. *Brain Pathol.* 22 219–229. 10.1111/j.1750-3639.2011.00520.x 21790829 PMC8029131

[B35] Peel A. L. Rao R. V. Cottrell B. A. Hayden M. R. Ellerby L. M. Bredesen D. E. (2001). Double-stranded RNA-dependent protein kinase, PKR, binds preferentially to Huntington’s disease (HD) transcripts and is activated in HD tissue. *Hum. Mol. Genet.* 10 1531–1538. 10.1093/hmg/10.15.1531 11468270

[B36] Poole S. Singhrao S. K. Kesavalu L. Curtis M. A. Crean S. (2013). Determining the presence of periodontopathic virulence factors in short-term postmortem Alzheimer’s disease brain tissue. *J. Alzheimers Dis.* 36 665–677. 10.3233/JAD-121918 23666172

[B37] Reimer L. Betzer C. Kofoed R. H. Volbracht C. Fog K. Kurhade C. (2021). PKR kinase directly regulates tau expression and Alzheimer’s disease-related tau phosphorylation. *Brain Pathol.* 31 103–119. 10.1111/bpa.12883 32716602 PMC8018097

[B38] Sandhu P. Naeem M. M. Lu C. Kumarathasan P. Gomes J. Basak A. (2017). Ser^422^ phosphorylation blocks human Tau cleavage by caspase-3: Biochemical implications to Alzheimer’s disease. *Bioorg. Med. Chem. Lett.* 27 642–652. 10.1016/j.bmcl.2016.11.08 27989667

[B39] Segev Y. Barrera I. Ounallah-Saad H. Wibrand K. Sporild I. Livne A. (2015). PKR inhibition rescues memory deficit and ATF4 overexpression in ApoE ε4 human replacement mice. *J. Neurosci.* 35 12986–12993. 10.1523/JNEUROSCI.5241-14.2015 26400930 PMC6605432

[B40] Shi C.-J. Peng W. Zhao J.-H. Yang H.-L. Qu L.-L. Wang C. (2020). Usnic acid derivatives as tau-aggregation and neuroinflammation inhibitors. *Eur. J. Med. Chem.* 187:111961. 10.1016/j.ejmech.2019.111961 31865017

[B41] Socransky S. S. Haffajee A. D. Cugini M. A. Smith C. Kent R. L. Jr. (1998). Microbial complexes in subgingival plaque. *J. Clin. Periodontol.* 25 134–144. 10.1111/j.1600-051x.1998.tb02419.x 9495612

[B42] Stancu I.-C. Cremers N. Vanrusselt H. Couturier J. Vanoosthuyse A. Kessels S. (2019). Aggregated tau activates NLRP3–ASC inflammasome exacerbating exogenously seeded and non-exogenously seeded tau pathology *in vivo*. *Acta Neuropathol.* 137 599–617. 10.1007/s00401-018-01957-y 30721409 PMC6426830

[B43] Tang Z. Liang D. Cheng M. Su X. Liu R. Zhang Y. (2021). Effects of *Porphyromonas gingivalis* and its underlying mechanisms on Alzheimer-like tau hyperphosphorylation in Sprague-Dawley rats. *J. Mol. Neurosci.* 71 89–100. 10.1007/s12031-020-01629-1 32557144

[B44] Urnowey S. Ansai T. Bitko V. Nakayam K. Takehara T. Barik S. (2006). Temporal activation of anti- and pro-apoptotic factors in human gingival fibroblasts infected with the periodontal pathogen, *Porphyromonas gingivalis*: potential role of bacterial proteases in host signalling. *BMC Microbiol.* 6:26. 10.1186/1471-2180-6-26 16524480 PMC1431544

[B45] Wu Z. Ni J. Liu Y. Teeling J. L. Takayama F. Collcutt A. (2017). Cathepsin B plays a critical role in inducing Alzheimer’s disease-like phenotypes following chronic systemic exposure to lipopolysaccharide from *Porphyromonas gingivalis* in mice. *Brain Behav. Immun.* 65 350–361. 10.1016/j.bbi.2017.06.002 28610747

[B46] Yoshiyama Y. Higuchi M. Zhang B. Huang S.-M. Iwata N. Saido T. C. (2007). Synapse loss and microglial activation precede tangles in a P301S tauopathy mouse model. *Neuron* 53 337–351. 10.1016/j.neuron.2007.01.010 17270732

[B47] Zhang J. Yu C. Zhang X. Chen H. Dong J. Lu W. (2018). *Porphyromonas gingivalis* lipopolysaccharide induces cognitive dysfunction, mediated by neuronal inflammation via activation of the TLR4 signaling pathway in C57BL/6 mice. *J Neuroinflammation* 15:37. 10.1186/s12974-017-1052-x 29426327 PMC5810193

